# Morphogenetic and physiological effects of LED spectra on the apical buds of *Ficus carica* var. Black Jack

**DOI:** 10.1038/s41598-021-03056-7

**Published:** 2021-12-08

**Authors:** Ankita Rajendra Parab, Kho Ying Han, Bee Lynn Chew, Sreeramanan Subramaniam

**Affiliations:** 1 School of Biological Sciences, Universiti Sains Malaysia (USM), 11800 Georgetown, Penang Malaysia; 2 Chemical Centre Biology (CCB), Universiti Sains Malaysia (USM), 11900 Bayan Lepas, Penang Malaysia; 3 School of Chemical Engineering Technology, Universiti Malaysia Perlis (UNIMAP), 02600 Arau, Perlis Malaysia; 4 National Poison Centre, Universiti Sains Malaysia (USM), 11800 Georgetown, Penang Malaysia

**Keywords:** Developmental biology, Plant sciences

## Abstract

The use of artificial light sources such as light-emitting diodes (LEDs) has become a prerequisite in tissue culture studies to obtain morphogenetic enhancements on in vitro plants. This technology is essential for developmental enhancements in the growing plant cultures due to its light quality and intensity greatly influencing the in vitro growing explants at a cellular level. The current study investigates the effects of different light-emitting diode (LED) spectra on the growth of apical buds of *Ficus carica* var. Black Jack. *Ficus carica,* commonly known as figs is rich in vitamins, minerals, and phytochemicals capable of treating microbial infections and gastric, inflammatory, and cardiac disorders. Apical buds of *Ficus carica* var. Black Jack, presented morphogenetic changes when grown under six different LED spectra. The highest multiple shoots (1.80 per growing explant) and healthy growing cultures were observed under the blue + red LED spectrum. Wound-induced callus formation was observed on apical buds grown under green LED spectrum and discolouration of the growing shoots were observed on the cultures grown under far-red LED spectrum. Multiple shoots obtained from the blue + red LED treatment were rooted using 8 µM indole-3-acetic acid (IAA), and the rooted plantlets were successfully acclimatised. Compared with the other monochromatic LEDs, blue + red proved to be significantly better for producing excellent plant morphogeny. It is apparent that blue and red LED is the most suitable spectra for the healthy development of plants. The findings have confirmed that the combination of blue + red LED can potentially be used for enhancing growth yields of medicinally and commercially important plants.

## Introduction

In the due course of evolution, plants have developed sensitivity towards a different spectrum of light for photosynthesis. Plants have varied morphological reactions when exposed to different light spectrums depending on the quantity of light absorbed by the photoreceptors in plants such as phytochromes, cryptochromes, and phototropins^1^. Traditional horticultural practices used illumination systems which included the usage of lamps of high-pressure sodium (HPS), metal-halide (MH), and mercury vapour (HPMV). Modern agricultural techniques which include artificial sources of light have become very popular and contribute to commercial success in crop improvement and agricultural practices^2,3^.

In vitro plant regeneration systems generally use artificial light systems to grow plants in laboratory conditions. Such artificial light systems consist of high-pressure sodium or fluorescent lamps with a wide range of wavelengths ranging from 350 to 750 nm^4^. The wide range of light wavelengths available in such lighting systems poses a limitation for the plant photosynthetic system in terms of absorption of specific light for growth^3,5–7^. A disadvantage of a high amount of electricity consumption and heat production is also a significant limitation to this practice. Thus installation of cooling systems becomes critical in such situations. This contributes to the unnecessary expenditure of time and resources^3,8–11^.

Grow light spectrum is a phenomenon that includes the absorption of a specific spectrum of light wavelengths by plants to undergo photosynthesis. Such light helps to enhance plant growth characteristics^12^. The light wavelength of 400–700 nm is considered the photosynthetically active radiation (PAR). The number of photosynthetically active photons falling at a given surface area or the photosynthetic photon flux density (PPFD) will determine the PAR absorbed by the plants^13–15^.

Thus, it is necessary to introduce such an artificial light system that has high photoelectric efficiency. Light-emitting diodes (LEDs) are potentially novel artificial light systems used in controlled agricultural and in vitro regeneration practices. LEDs' significance had been noted in terms of long life, non–heating, cost-effectiveness, reduced size, and reduced energy consumption. When assigned to the LEDs, a specific band wavelength will increase the probability of growth and development of desirable traits^11,14^. Generally, plants absorb the red light spectrum from the standard white light for enhanced photosynthetic ability because the wavelength for red light (660 nm) is near the chlorophyll pigment's absorption wavelength.

Along with the red light spectrum, the blue light spectrum also plays a vital role in photomorphogenesis in plants^11^. It was reported that red light and some blue light percentages would produce optimal plant growth^14–16^. Plants grown under the far-red LEDs showed underdeveloped leaves^8^. Mills and Dunn^9^, reported that different plants have specific responses to specific light intensities for example, on the seeds of lettuce (*Lactuca sativa* L.) the red light (660 nm) produced elongated hypocotyls. In contrast to the strawberry plant (*Fragaria ananassa* D.), it produced an enhanced photosynthesis rate. Red (660 nm) + blue (470 nm) light also produced an increased rate of photosynthesis in the rice plants (*Oryza sativa* L.). Red + white (600 nm – 700 nm) light stunted the growth of the Ageratum (*Ageratum houstonianum* 'Hawaii Blue') and calibrachoa (Calibrachoa x hybrida 'Callie White') hybrid plant.

*Ficus carica* L. (fig) is one of the oldest species of fruit-bearing trees, consisting of over 800 species and thousands of varieties, and is traditionally cultivated for its medicinal properties^17^. Fig (*Ficus carica* L.) is rich in flavonoids, phenols, and other medicinally essential biochemicals. Traditionally fig trees are used to cure gastric, inflammatory, diabetic, bacterial infections, and cardiovascular ailments. Among many varieties, 'Black Jack' fig variety is trending in the Asian subcontinent. This variety's edible fruits are large and bear a darker brown to purple colour^18,19^. With the increasing demand for fig cultivation application of advanced micropropagation technologies to enhance the yields is essential. Thus mass cultivation and enhancement of the fig species have become a priority^20–23^.

The use of LED for micropropagation studies, compared with other light sources, has exceptional advantages. Thus advancement of research for plant tissue culture using different LEDs as the source of light for the enhanced growth and development of in vitro grown plants is a very convenient and practical technique. Greater quantum efficiency is offered by LEDs, enhancing the morphological and physiological properties of the in vitro growing plants. Thus for many tissue culture-based projects market plant-specific LEDs^24^.

Increasing interest in the use of LEDs for plant growth and development has aroused recently. Thus this experiment aims to check the morphogenetic and physiological effects of six different wavelengths of LED spectra on the apical buds of *Ficus carica* var. Black Jack. Based on prior studies, it has been hypothesized that blue and red LED should potentially improve the growth of apical buds of *Ficus carica* var. Black Jack.

## Materials and methods

### Plant materials

Mother plants of *Ficus carica* var. Black Jack were cultivated in farms, by ‘Fig Direct’, Superfruit Valley, Perlis. The most effective explant used for the in vitro propagation is the apical buds excised from the apex of the shoot (or shoot tip). Healthy apical buds were collected from 2 year-old mature mother plant of *Ficus carica* var. Black Jack, which was maintained in the School of Biological Sciences, Universti Sains Malaysia. The explants (apical buds) were initially washed and brushed with water, and then thoroughly rinsed under running tap water for 30 min, followed by a robust sterilisation protocol. The sterilisation protocol included sequentially washing the explants in 70% Ethanol, and 50% Clorox + 2 drops of tween 20, for 10 min each. This step was repeated twice, followed by washing the apical buds with sterile distilled water (8–10 changes) for each between 2–3 min. Apical buds were then cultured and grown on Woody Plant Media (WPM) supplemented with 20 µM BAP and 8 µM IAA. WPM was used as the basal media to induce and proliferate multiple shoots on the apical buds of *Ficus carica* var. Black Jack (McCown Woody Plant Media^25^-DUCHEFA; 2.46 g/L) along with sucrose (15 g/L) for shoot induction from the apical bud explants. Gelrite was used as a gelling agent, 3 g/L. The pH of all media was adjusted between 5.7 and 5.8 prior to autoclaving at 1.05 kg/cm2, 121 °C for 20 min. After culturing the ~ 0.4 cm apical bud on the WPM, the i*n vitro* cultures were incubated at 25 ± 1 °C under white fluorescent light (Philips TLD, 36 W, 60 μmol.m^-2^ s^-1^) for a daily 16-h photoperiod.

### Preparation of the explant

In vitro regenerated plants of *Ficus carica* var. Black Jack was used in this study. The most commonly used explants for the following experiments were the apical buds (from the apex of the in vitro shoots) of in vitro plants. In vitro plant of *Ficus carica* var. Black Jack has grown for almost 4 -5 weeks, on woody plant medium (WPM)^25^, supplemented with 6-benzylaminopurine (BAP) and indole-3-acetic acid (IAA) (WPM + 20 µM BAP + 8 µM IAA) as basal media solidified with Gelrite. Apical buds were excised from these in vitro plants and cultured on media containing the optimised plant growth regulator (PGR) (20 µM BAP).

### Culture conditions

Woody plant medium (WPM) [McCown Woody Plant Medium^25^-DUCHEFA; 2.46 g/L] was used as the basal media along with sucrose (15 g/L) for shoot induction from the apical bud explants. Gelrite was used at 3 g/L. The PGR (BAP) was purchased from DUCHEFA, Netherlands. The pH of medium was adjusted between 5.7 and 5.8 prior to autoclaving at 1.05 kg/cm^2^, 121 °C for 20 min. The LED spectra were selected for growing the excised apical buds of *Ficus carica* var. Black Jack is shown in Table 1. Apical buds were cultured on WPM supplemented with 20 µM BAP and the six different treatments are shown in Table 2.

**Table 1:** Light intensity and spectral power distribution (SPD) of the LED treatments on the apical buds of *Ficus carica* var. Black Jack.

Light Sources	Light intensity (µmol m^-2^ s^-1^)	Spectral Power Distribution (SPD)
White LED (400 nm–700 nm)	17.0	
Blue (440 nm)	15.7	
Green (550 nm)	16.9	
Red (660 nm)	15.4	
Far-red LED (725 nm)	20.8	
Blue + Red (440 nm + 660 nm) (1:1)	20.3

**Table 2:** Effects of different LED spectra for shoot induction on the apical buds of *Ficus carica* var. Black Jack after 20 weeks.

Treatment	LED spectra	No. of shoots (*x̄*)	Survival (%)	Height (*x̄*) (cm)	No. of leaves per single shoot (*x̄*)	Callus (%)	Browning (%)
T1	White (400–700 nm)	1.47 ± 0.83^e^	87	0.80 ± 0.91^cd^	2.00 ± 1.05^cd^	0	0
T2	Blue (440 nm)	1.20 ± 1.94^abcde^	40	0.72 ± 0.91^cd^	1.50 ± 1.90^bc^	0	0
T3	Green (550 nm)	0^cde^	0	0^d^	0^d^	100	0
T4	Red (660 nm)	1.73 ± 4.00^abcde^	33	1.08 ± 1.59^ab^	1.93 ± 2.87^abc^	0	35
T5	Far-red (725 nm)	0^ab^	0	0^d^	0^d^	0	0
T6	Blue + red (440 nm + 660 nm) (1:1)	1.80 ± 2.37^a^	60	1.20 ± 1.32^a^	3.00 ± 2.95^a^	0	0

^a^Means (x̄) followed by the same letter within a column were not significantly different using Tukey test (*P* ≥  0.05).

^b^Media used to grow the apical buds under different LED treatments (T1–T6) was WPM + 20 µM BAP.

^c^Callus (%) indicates the frequency of callus formed on a single apical bud explant when grown under different LED treatments.

^d^Browning (%) indicates the browning of the tips of the multiple shoots formed on the apical buds, after 5th subculture.

### Effects of different LED spectra on shoot induction

After culturing the ~ 0.4 cm apical bud on the WPM, in vitro cultures were incubated at 25 ± 1 °C under 6 different LED treatments namely white (400–700 nm), blue (440 nm), green (550 nm), red (660 nm), far-red (725 nm), and blue + red (440 + 660 nm) (Table 1) for a daily 16-h photoperiod. The cultures were subcultured every 4 weeks for 5 subculture cycles.

### Induction of in vitro root culture

Roots were induced on the acquired multiple shoots plants grown under blue + red LED. For the induction of roots, different concentrations of IAA were used (Table 3). WPM supplemented with 20 µM of BAP was used as the basal media with different IAA concentrations to induce roots. The PGRs used for this study were purchased from DUCHEFA, Netherlands. Media was solidified using Gelrite (3 g/L). The pH was adjusted between 5.7 and 5.8 with NaOH or HCl before autoclaving the media at 121 °C for 15 min (STURDY SA-300VFA-F-A505, Sturdy Industrial Co. Ltd., Taiwan). Glass jars containing 40 mL of media were used to grow the plantlets. Cultures were incubated at 25 ± 1 °C under blue + red LED for a daily 16-h photoperiod.

**Table 3:** Effects of different IAA concentrations on the induction of roots, on the single shoots obtained from cultures under blue + red LED.

Treatment	BAP (µM)	IAA (µM)	No. of Roots (*x̄*)	Rooted plantlets (%)
R1	20	2	1.67 ± 0.16^b^	100
R2	20	4	1.67 ± 0.19^b^	100
R3	20	6	1.87 ± 0.17^b^	100
R4	20	8	2.73 ± 0.23^a^	100
R5	20	10	1.93 ± 0.21^b^	100

^a^Means (x̄) followed by the same letter within a column were not significantly different using Tukey test (*P* ≥ 0.05).

^b^Rooted plantlets (%) indicates the total percentage of plantlets successfully rooted under blue + red LED spectra.

### Acclimatisation of the in vitro regenerated plants

Plantlets with well–developed roots were used for the acclimatisation process. The plantlets were removed from the jar and washed under tap water and transferred to plastic trays containing sterile soil (BioChar Soil Mix 1, Serbajadi). The process of acclimatisation was adapted according to Hazarika et al.^26^. In vitro plants were transferred in 4 × 4 small pots containing sterile BioChar soil for 2 weeks before gradually transferring the plants in larger pots in the greenhouse. The *ex vitro* acclimatised plants were regularly monitored and watered. This method of acclimatisation is used to produce disease-free micropropagated plants of *Ficus carica* var. Black Jack^26^.

### Statistical analysis

The design for all experiments was completely randomised. The results were expressed as mean ± standard error (SE). The mean values were subjected to a one-way analysis of variance (ANOVA). Tukey's multiple range post hoc test was done to determine the significance at *p* ≤ 0.05. All data analysis was carried out using IBM SPSS version 26.0.

### Apical buds growing under six different LED spectra

Each culture jar consisted of five explants initially, and a total of thirty cultures were grown under each treatment. The data, such as the number of shoots was recorded after every 4 weeks of culturing. After 4 weeks the cultures were subcultured to new media. Subculture was repeated 5 times (over 20 weeks), and the data was recorded for each subculture.

### Rooting of the multiple shoots growing under blue + red LED

Three (3) explants were cultured in one jar and ten (10) cultures were raised in each treatment. The number of roots was determined after 8 weeks of culturing.

## Results and discussion

### Effects of different LED spectra on the growth of apical buds of *Ficus carica* var. Black Jack

The photochemical activities in plants that are controlled by chlorophyll are positively affected by the use of LEDs. The in vitro and *ex vitro* plants exhibit different photosynthetic photon flux density (PPFD) or absorption capacities of the light photons depending on the light's availability and the photoreceptors present in the plants. The quality and quantity of light and endogenous phytohormones are responsible for guiding the overall morphological changes in the in vitro growing plants^6,24^. The effects of six different LED spectra were studied on the growth of apical buds acquired from in vitro plants of *Ficus carica* var. Back Jack.

### Effects of green LED on the growth of apical buds

**Figure 1:**
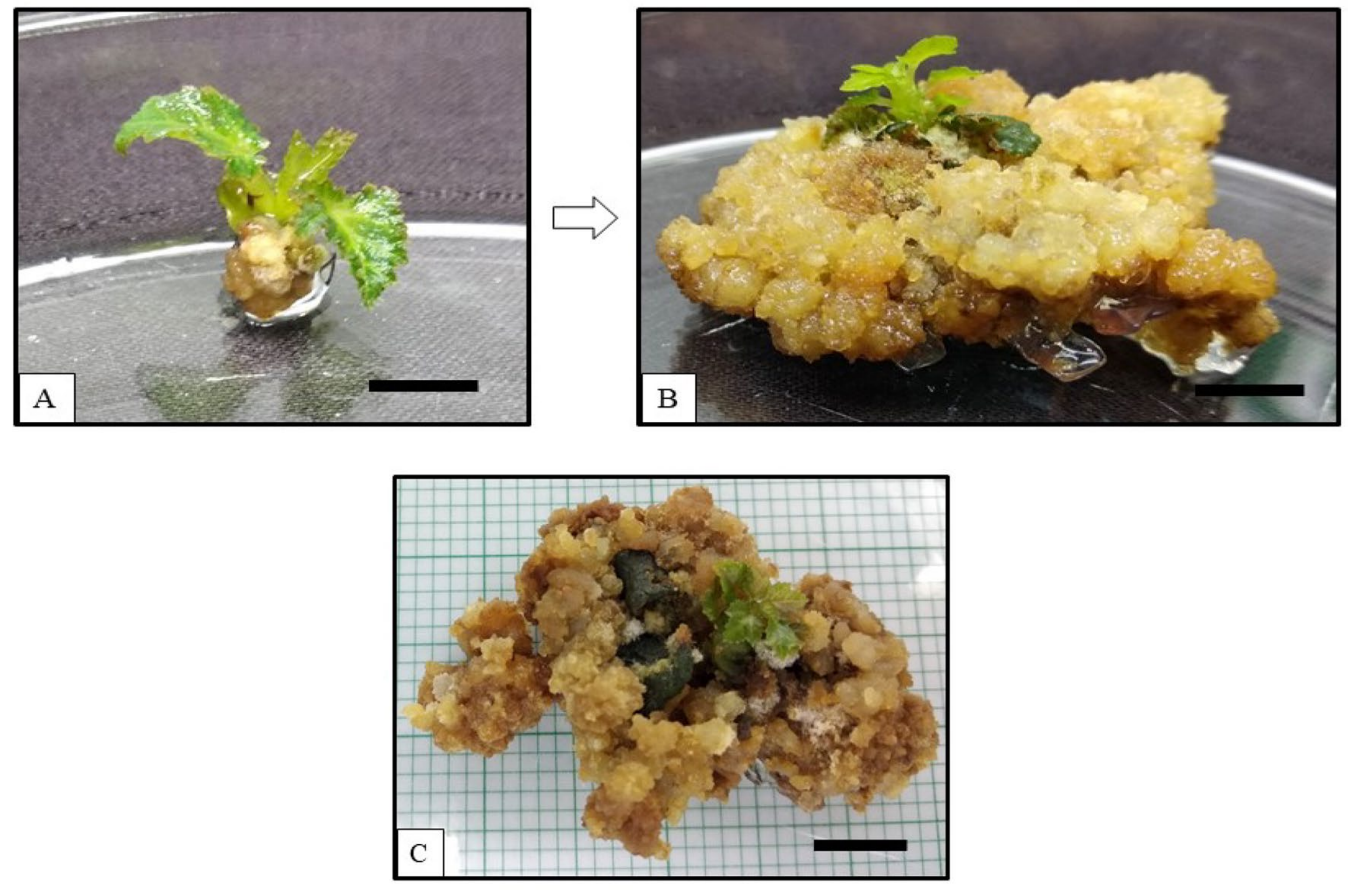
Effects of green LED on the apical buds of *Ficus carica* var. Black Jack. (**A**) Apical bud break and induction of callus formation were observed after 2 weeks of culture, and (**B**) Callus proliferation was observed after 8 weeks of culture and (**C**) Browning of callus and no multiple shoots were observed after 12 weeks. Scale bar represents 1 cm.

Heavy callus induction was observed on the apical buds growing under green LED produced callus, which reduced the formation of the shoot (Fig. 1). After 2 weeks of growth under green LED, callus formation was observed around the apical bud base (Fig. 1A), which progressed after 8 weeks (Fig. 1B). After 8 weeks, the cultures started browning and finally terminated after 12 weeks (Fig. 1C). Unorganised mass of plant cells is clumped together and forms a callus. Callus induction in plants is a response to many biotic and abiotic stresses. The abiotic factors responsible for such a response include the exogenous application of plant growth regulators (PGRs) and in vitro growth conditions. A wound-induced callus responds to any surface wounds on the growing explants, resulting from positive biosynthetic pathways followed by the cytokinins^27^. Thus no significant multiple shoot data were obtained from cultures grown under green LED.

Studies conducted on *Cunninghamia lanceolata* (Lamb.) Hook. Includes the application of a combination of red, blue, purple, and green LEDs on the growing explants. The effects of the different LEDs were observed on the growing cultures, and it was reported that the requirement for green light is lower than the other LEDs^15^. Plants growing under green light showed two drastically opposite (antagonistic) responses towards plant growth. The development of green light antagonistic effects suppressed culture growth. The low intensity of 16.9 µmol m^−2^ s^−1^ of green LED, decreased photosynthetic activity by reducing specific photons' directional flow. This overall contributed to the inhibition of plant growth and development, followed by the formation of callus. The growth of multiple shoots on the apical buds was negatively influenced by reducing the PPFD of the apical buds. The capacity for photoabsorption under green light by chlorophyll is also deficient. Therefore, the cascade mechanism of wound-induced callus formation was activated on the plantlets growing under the green LED spectrum, which contributed to the underdevelopment of plantlets^28,29^.

### Effects of far-red LED on the growth of apical buds

**Figure 2:**
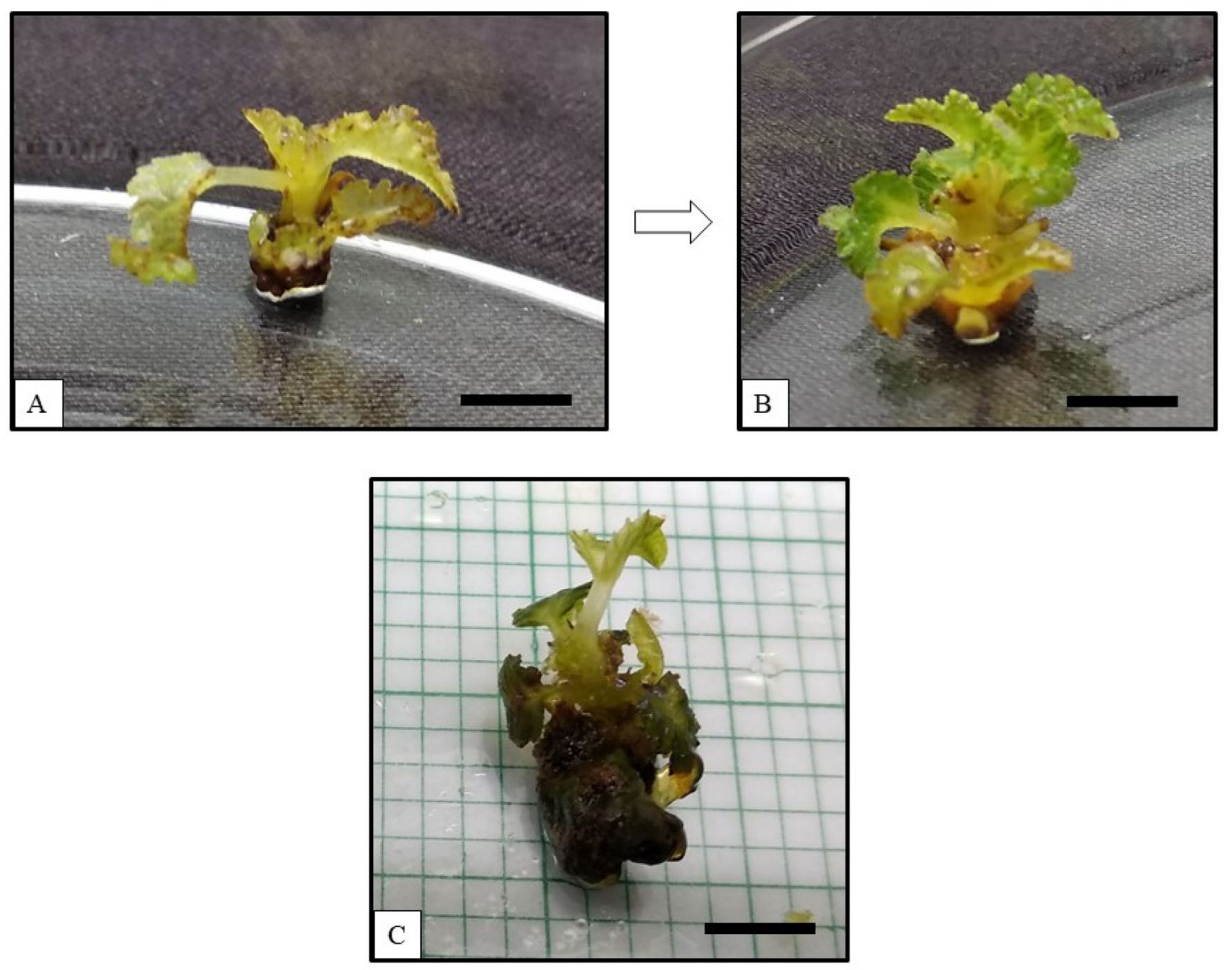
Effects of far-red LED on the apical buds of *Ficus carica* var. Black Jack. (**A**) The absence of apical bud break and discolouration of the apical bud was observed after 2 weeks of culture, (**B**) 2–3 multiple shoots, and discolouration was observed after 8 weeks of culture, and (**C**) Multiple shoot formation was ceased and culture did not survive after 12 weeks. Scale bar represents 1 cm.

Apical buds growing under far-red LED are shown in Fig. 2. These cultures were also terminated post 12 weeks. No bud break was observed on the apical buds post 2 weeks of culture. However, discolouration of the apical buds was observed throughout (Fig. 2A). A total of 2–3 multiple shoots was obtained on the growing apical buds (Fig. 2B). The unhealthy shoots growing under far-red LED after 12 weeks, Fig. 2C were terminated eventually. Survival of apical buds post 20 weeks, was observed to be 0% and no significant data was noted (Table 2).

Far-red LED did not possess any wavelength of the blue LED and the available intensity of the far-red LED was 20.8 µmol m^−2^ s^−1^. Discolouration of the apical bud explants with lesser green pigmentation and weaker stems in the growing cultures can be attributed to the reduced chlorophyll pigment. Eventually, the cultures underwent senescence and died. Far-red light of the wavelength 730 nm was reported to be successful for flowering and overall growth in plants. Plants absorb blue light to increase the chlorophyll content, enhancing the overall development of the plants^9^. Therefore, the absence of the blue wavelength contributes towards the cultures under far-red LED being terminated. Thus, photosynthesis in plants can be carried out efficiently in blue and red light in an equal ratio^11,30^.

### Effects of white, blue, red and blue + red LEDs

**Figure 3:**
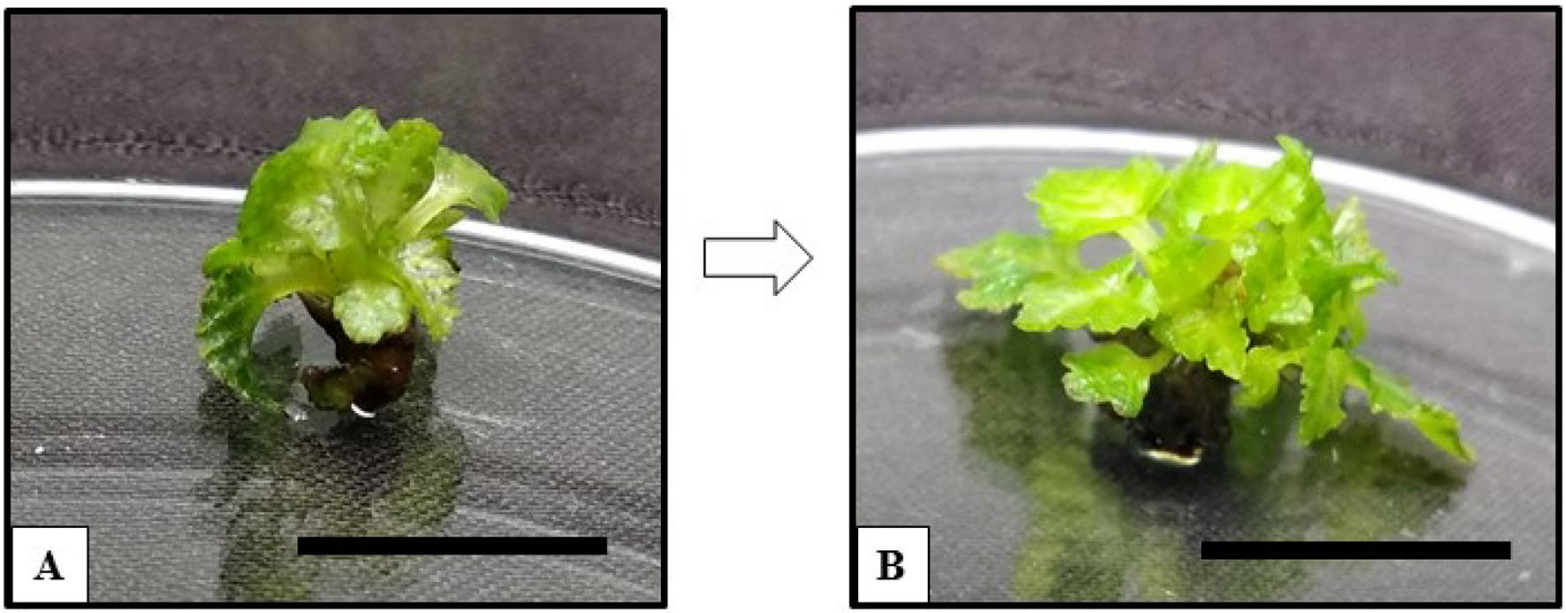
Effects of blue + red LED spectrum on the apical buds of *Ficus carica* var. Black Jack. (**A**) Apical bud break observed after 2 weeks of culture, (**B**) multiple shoot induction observed after 8 weeks of culture. Scale bar represents 1 cm.

Apical buds, growing under the rest of the four LEDs showed bud break at 2 weeks, and approximately 2 to 4 multiple shoots were observed after 8 weeks of the growing cultures (Fig. 3).

### Effects of white, blue and red LEDs

**Figure 4:**
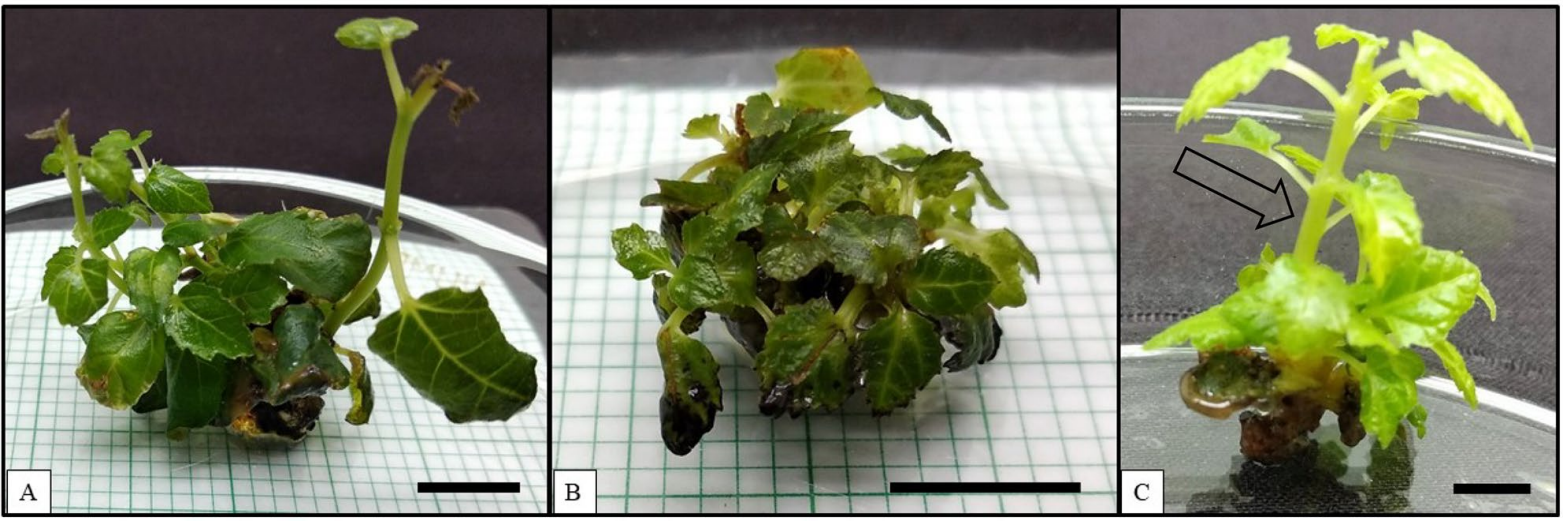
Effects of white, blue and blue + red LED spectra on the apical buds of *Ficus carica* var. Black Jack. (**A**) Multiple shoots produced on the apical buds after 20 weeks of culture in white LED, (**B**) Multiple shoots produced on the apical buds after 20 weeks of culture in blue LED, and (**C**) Multiple shoots produced on the apical buds after 20 weeks of culture in red LED. Scale bar represents 1 cm.

Multiple shoots were observed on apical buds growing under white, blue, and red LEDs. The LEDs offer a targeted flow of photons towards the growing cultures. This is a major difference between the normal fluorescent white light (60 μmol m^−2^ s^−1^) and white LED (17 μmol m^−2^ s^−1^). Although the photosynthetically active radiation (PAR) wavelengths offered by both the lights are similar, the light intensity of white LED is lower than the normal fluorescent white light^6,28^. Therefore, the average number of shoots observed in cultures grown under the white LED was only 1.47 (Fig. 4A; Table 2) shoots per growing explant.

Cultures growing under blue LED showed 40% survival with an average number of shoots observed at approximately 1.20 shoots per growing apical bud (Fig. 4B; Table 2). The average height of each shoot was approximately noted at 0.72 cm with a 1.50 number of leaves on an average on the single shoot. Plantlets growing under blue LED were observed to have stunted growth (Fig. 4B).

Plant cultures of *Ficus carica* var. Black Jack growing under the red LED is observed in Fig. 4C. At 16 weeks, cultures growing under red LED produced a higher number of multiple shoots (1.73 per growing apical bud) (Table 2). Stem elongation was observed on the single shoots and after 16 weeks of culture, the multiple shoots did not elongate further, and no additional multiple shoots were obtained (Fig. 4C). The average height was approximately 2.5 cm and the number of leaves per single shoot was approximately 4.5 (Table 2).

**Figure 5:**
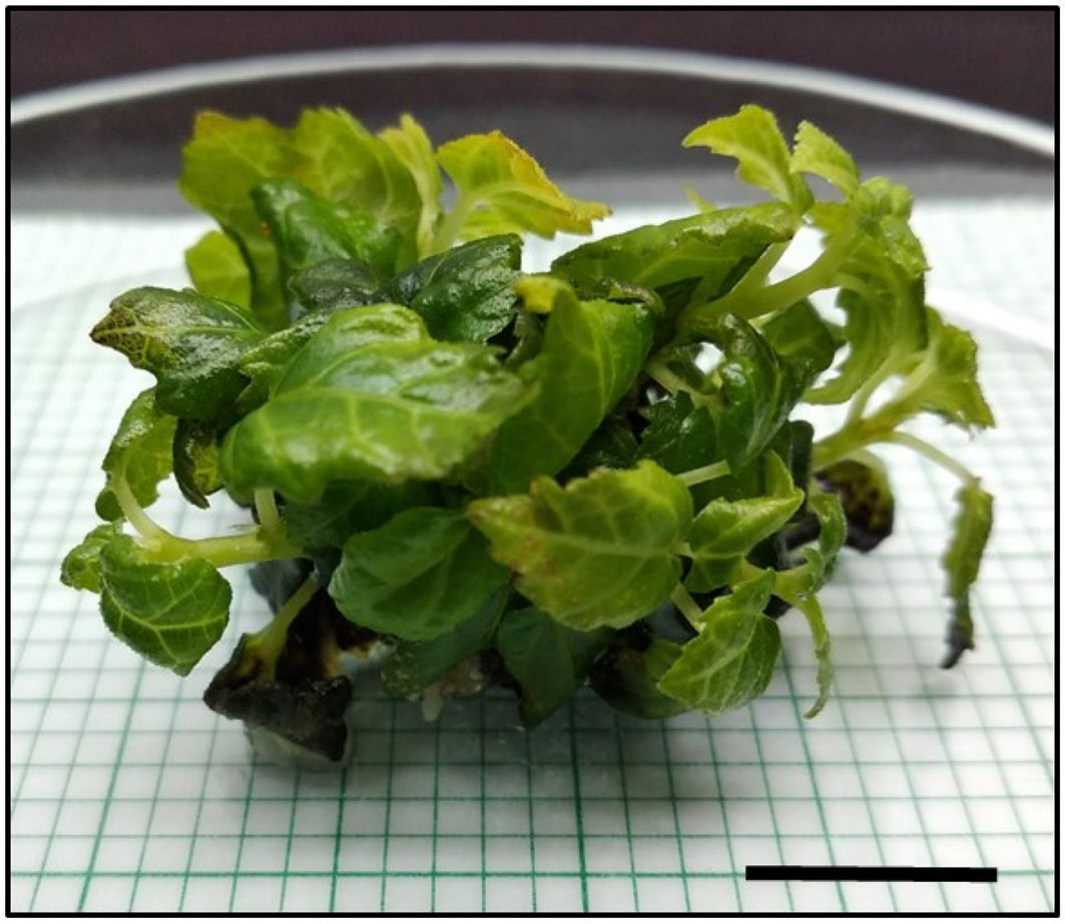
Effects of blue + red LED spectrum on the apical buds of *Ficus carica* var. Black Jack. Multiple shoots were produced on the apical buds after 20 weeks of culture. Scale bar represents 1 cm.

Higher efficiency for in vitro plant growth cannot be observed using a monochromatic blue LED^7^, stunted growth was observed among the tomato cultivars grown under blue light. A similar response was observed for growing cultures of *Ficus carica* var. Black Jack. Despite the cultures producing multiple shoots, the shoots' height was shorter than the plants growing in the blue + red LED (Figs. 4 and 5; Table 2). Decreased plant height was also observed in Petunia hybrid plants (*Petunia atkinsiana* Juss.) under red light. In some cases, blue light was noted to produce an enhanced biochemical yield in plants by increasing the plant's chlorophyll pigments. Also, red LED had produced shoots on the explant in some plants, Despite these facts, monochromatic blue and red lights did not produce enhanced responses among the many other growing plantlets^1,7,30^.

A red light positively influences photosynthetic activity. For example, a higher number of shoots were induced when lettuce was grown under red light^5^. Thus, stem elongation can be attributed to enhanced photosynthetic activity. This ultimately resulted in plant growth rather than multiplication. There is an absence of blue LED photons in the monochromatic red LED, which resulted in discolouration of the in vitro plants. The data for an average number of shoots obtained for plantlets growing under white, blue, and red LEDs were significantly lower than the data obtained from cultures grown under blue + red LEDs. The effects of the combination of blue and red LED wavelengths surpassed the other LEDs^12,29^.

### Effects of blue + red LED

The combination of the blue and the red light mixture had been reported to be highly efficient for the growth and development of in vitro plants. The yield of lettuce plants (*Lactuca sativa* L.) was observed to be higher when grown under blue and red LED light^5^. Similar, results were obtained on plants such as *Cannabis sativa* L. cultivar, 'Pak Choi' (*Brassica rapa* L.), and 'Tomato' (*Solanum lycopersicum* L.), and *Lactuca sativa* L. cv. 'Fire Red'^1,11,30^. Plant photoreceptors can absorb a specific spectrum of light to induce organogenesis. In the monochromatic LED spectrum systems, the PPFD photoreceptors are adjusted to match the photosynthetically active radiation (PAR) of the in vitro plants. This reduces the risk of the growing culture chambers in terms of overheating. The use of LED spectra ensures a linear flow of the photon towards the growing plant cultures^6^.

The survival rate for the cultures growing under blue + red LED was 60%, and blue + red LED on average produced a significantly high number of shoots on the apical bud at 1.80 (Fig. 5, Table 2) compared with the other LEDs. The highest shoot length observed was 1.20. The average number of leaves on every single shoot was observed to be 3.00 leaves per single shoot (Table 2). Morphologically healthier plants were obtained from cultures grown under blue + red LED than the cultures growing under white and blue LEDs. The leaf colour of cultures growing in blue + red LEDs was observed to be darker than the leaves of cultures grown in the white and blue LEDs (Figs. 4 and 5).

The current experiment used blue + red LED spectra in the ratio 1:1, which was revealed to be the best for growing healthy plants of *Ficus carica* var. Black Jack. This was attributed to the available 20 µmol m^-2^ s^-1^ light intensity and targeted monochromatic lights of blue (440 nm) and red (660 nm) LEDs (Table 1). For most plant species, the ideal photon absorbance (PPDF) is 30 – 40 µmol m^-2^ s^-1^^7^. The intensity for white LED (17 µmol m^-2^ s^-1^) was notably low, due to this, the apical buds produced a significantly lower number of multiple shoots. Thus plantlets of *Ficus carica* var. Black Jack grown under blue + red LED showed a higher number of multiple shoots than individual white, blue and red LEDs. Black Jack variety of figs (*Ficus carica* L.) are in high demand and currently trending in the Southeast Asian (SEA) markets^18,23,31,32^. The application of a combination of blue + red LEDs for growing healthy plantlets of *Ficus carica* var. Black Jack is a suitable technique to achieve multiple shoots and healthy growing plantlets. This would aid in the large-scale commercialisation of the currently trending fruit in the SEA markets.

### Induction of in vitro roots on the multiple shoots obtained from blue + red LED spectra

**Figure 6:**
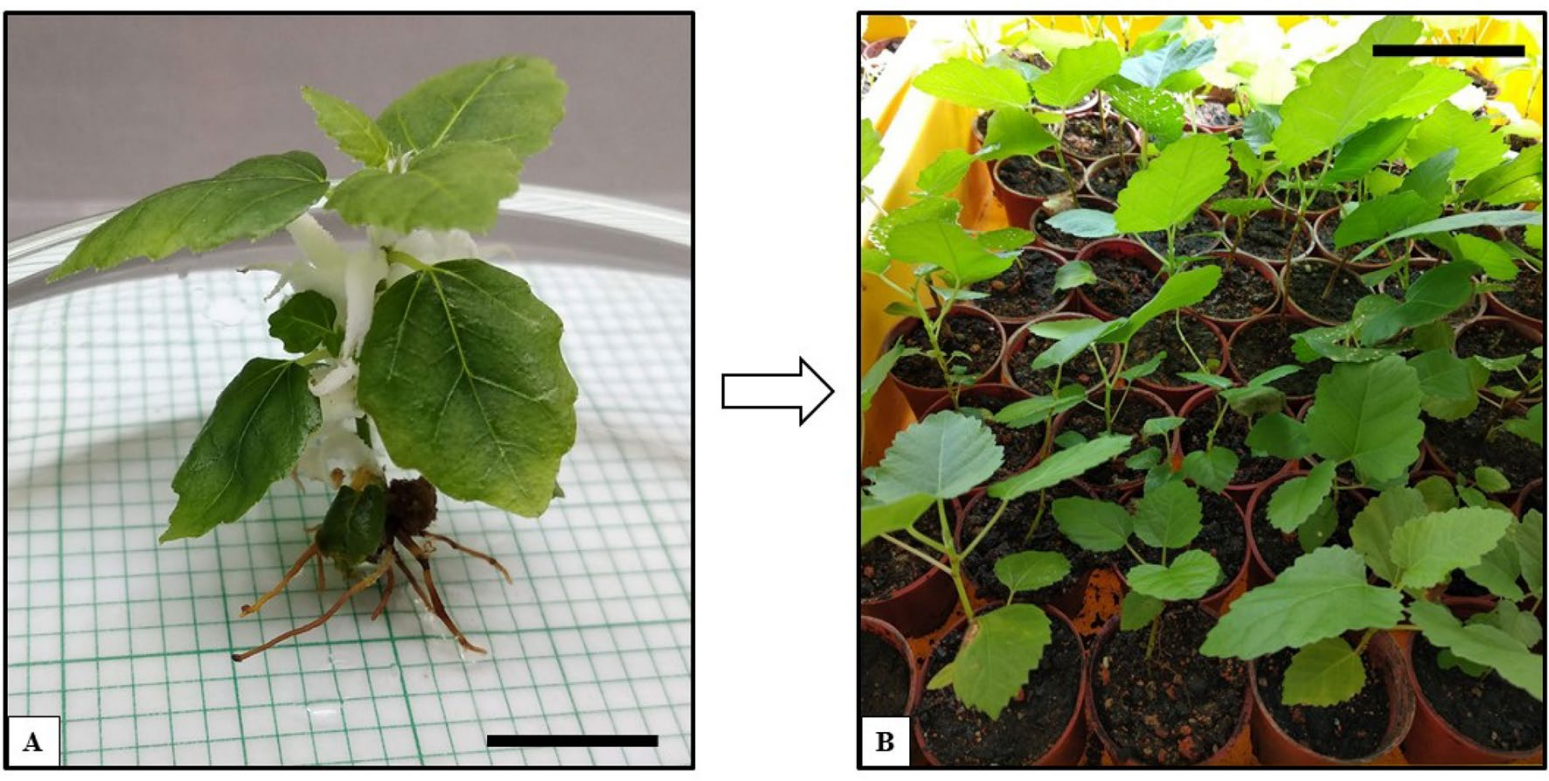
Rooted plantlet of *Ficus carica* var. Black Jack from single shoots growing under blue + red LED. (**A**) Four to six roots were observed on the growing shoot after 4 weeks of a subculture, on the R4 rooting media. Scale bar represents 1 cm and (**B**) Establishment of successful acclimatisation of plantlets grown under blue + red LED spectra of *Ficus carica* var. Black Jack. Scale bar represents 4 cm.

The effects of different IAA concentrations in WPM + 20 µM BAP were tested for the formation of roots. Multiple shoot was obtained from cultures of F*icus carica* var. Black Jack, growing under blue + red LED were used. Shoots with an average height of approximately 1.80 cm were transferred to the rooting media with different IAA concentrations (WPM + IAA) (Table 3). The highest number of roots were generated on media R4 (WPM + 20 µM BAP + 8 µM IAA). An average of 2.73 roots per shoot with 100% successful root induction was observed (Fig. 6A; Table 3).

Successful root induction using IAA was also observed on the in vitro grown plant cultivars of *Ensete ventricosum* Welw and *Vanilla planifolia* Andr.^33,34^. Similarly, multiple shoots induced on *Ficus religiosa* L. using BAP were rooted in a combination of BAP and IAA in MS media^35,36^. It is observed that the addition of cytokinin in the media promotes the rooting of the in vitro plants of *Cassia angustifolia* Vahl^6^. The effects of LEDs are different for every plant species^15^. It was reported that the combination of blue and red light together is essential for promoting photosynthetic activity and root induction in plants. This encourages the ideal growth and development of the plants. A combination of blue and red lights is reported to be beneficial for the overall efficacy of plant production^12^.

Rooted plants from the blue + red LED were used for *ex vitro* acclimatisation. Sterile BioChar Soil was used for the acclimatisation process. Rooted plantlets were transferred to small pots (4 cm × 4 cm) containing the sterile soil and incubated under white fluorescent light (Philips TLD, 36 W, 60 μmol m^-2^ s^-1^) for a daily 16-h photoperiod for two weeks before transferring to larger pots. After 8 weeks in the *ex vitro* conditions, the successfully acclimatised plants can be observed in Fig. 6B. The in vitro growth conditions of the plants create abiotic and biotic stress on the plantlets. Thus, plant anatomy and physiology may drastically vary from the *ex vitro* plants^26^.

## Conclusions

Multiple shoots induction was observed on the in vitro cultured apical buds of *Ficus carica* var. Black Jack under the white (400–700 nm), blue (440 nm), red (660 nm), and blue + red (440 + 660 nm) LED using WPM + 20 µM BAP. Blue + red LED used in the ratio 1:1 was reported to be the best to induce multiple shoots. The highest number of multiple shoots under this treatment was obtained at 1.80^a^. By using the combination of WPM medium supplemented with 20 µM BAP and 8 µM IAA, the highest number of roots were successfully induced on the in vitro cultures growing under blue + red LED. The rooted plants were acclimatised in the *ex vitro* conditions using sterile BioChar soil.

## Data Availability

Appropriate guidelines were followed for the use of plants in the current study. All data generated or analysed during this study are included in this published article.

## Abbreviations

BAP: 6-Benzylaminopurine
HPMV: High pressure-mercury vapour
HPS: High-pressure sodium
IAA: Indole-3-acetic acid
LED: Light-emitting diode
MH: Metal-halide
No.: Number
PAR: Photosynthetically active radiation
PGR: Plant growth regulator
PPFD: Photosynthetic photon flux density
WPM: Woody plant media

## References

[1] Lalge, A., Cerny, P., Trojan, V., & Vyhnanek, T. The effects of red, blue and white light on the growth and development of *Cannabis sativa* L. in *Mendel Net 2017 conference paper*, 646–651 (2017).

[2] Jiang N, Grundy S, Bian Z, Lu C (2015). Investigation of LED light effects on plant growth in improved protected horticulture system. J. Am. Soc. Hortic. Sci..

[3] Monostori I (2018). LED lighting–modification of growth, metabolism, yield and flour composition in wheat by spectral quality and intensity. Front. Plant Sci..

[4] Bidabadi SS, Mohan JS (2020). Cellular, molecular, and physiological aspects of *in vitro* plant regeneration. Plants.

[5] Borowski E, Michałek S, Rubinowska K, Hawrylak-Nowak B, Grudziński W (2015). The effects of light quality on photosynthetic parameters and yield of lettuce plants. Acta Scientiarum Polonorum Hortor. Cultus.

[6] Cioć M, Szewczyk A, Żupnik M, Kalisz A, Pawłowska B (2018). LED lighting affects plant growth, morphogenesis and phytochemical contents of *Myrtus communis* L. in vitro. Plant Cell Tissue Organ Cult..

[7] Cioć M, Kalisz A, Żupnik M, Pawłowska B (2019). Different LED light intensities and 6-benzyladenine concentrations in relation to shoot development, leaf architecture, and photosynthetic pigments of *Gerbera jamesonii* Bolus *In Vitro*. Agronomy.

[8] Massa GD, Kim HH, Wheeler RM, Mitchell CA (2008). Plant productivity in response to LED lighting. HortScience.

[9] Mills T, Dunn B (2016). LED grow lights for plant production. Oklahoma Coop. Ext. Serv..

[10] Hasan MM, Bashir T, Ghosh R, Lee SK, Bae H (2017). An overview of LEDs’ effects on the production of bioactive compounds and crop quality. Molecules.

[11] Bian Z, Jiang N, Grundy S, Lu C (2018). Uncovering LED light effects on plant growth: New angles and perspectives–LED light for improving plant growth, nutrition and energy-use efficiency. Acta Horticulturae.

[12] Bios. The ideal LED grow light spectrum for plants. Preprint at https://bioslighting.com/horticulture-lighting/grow-light-spectrum-led-plants/ (2020).

[13] Olle M, Viršile A (2013). The effects of light-emitting diode lighting on greenhouse plant growth and quality. Agricult. Food Sci..

[14] Park Y, Runkle ES (2018). Spectral effects of light-emitting diodes on plant growth, visual color quality, and photosynthetic photon efficacy: white versus blue plus red radiation. PLoS ONE.

[15] Xu Y, Liang Y, Yang M (2019). Effects of composite LED light on root growth and antioxidant capacity of *Cunninghamia lanceolata* tissue culture seedlings. Sci. Rep..

[16] Poudel PR, Kataoka I, Mochioka R (2008). Effect of red- and blue-light-emitting diodes on growth and morphogenesis of grapes. Plant Cell Tissue Organ Cult..

[17] Singh A, Prakash J, Meghwal PR (2015). Fig (*Ficus carica* L.). Breed. Underutil. Fruit Crops.

[18] Wongmetha, O. A Study on Fig Varieties in Northern Thailand. International Sub-Tropical Workshop China. Preprint at https://www.researchgate.net/publication/331009693_a_study_on_fig_varieties_in_northern_thailand. (2008).

[19] Vinson, E. Fig Production Guide. Alabama Cooperative Extension System. Preprint at https://www.aces.edu/wp-content/uploads/2019/08/anr-1145_figproductionguide_080919l-g.pdf. (2019).

[20] Al-Yousuf HHH (2012). Antibacterial activity of *Ficus carica* L. extract against six bacterial strains. Int. J. Drug Dev. Res..

[21] Ahmad ZM, Ali M, Mir SR (2013). Anti-diabetic activity of *Ficus carica* L. Stem barks and isolation of two new flavonol esters from the plant by using spectroscopical techniques. Asian J. Biomed. Pharm. Sci..

[22] Abdel-Aty AM, Hamed MB, Salama WH, Ali MM, Fahmy AS, Mohamed SA (2019). *Ficus carica*, *Ficus sycomorus* and *Euphorbia tirucalli* latex extracts: phytochemical screening, antioxidant and cytotoxic properties. Biocatal. Agricult. Biotechnol..

[23] Shamin-Shazwan K, Shahari R, Che Amri CNA, Tajuddin NSM (2019). Figs (*Ficus carica* L.): cultivation method and production based in Malaysia. Eng. Herit. J..

[24] Olvera-Gonzalez E (2014). A LED-based smart illumination system for studying plant growth. Light. Res. Technol..

[25] McCown BH, Lloyd G (1981). Woody Plant Medium (WPM)—a mineral nutrient formulation for microculture of Woody plant species. HortScience.

[26] Hazarika BN, da Teixeira SJA, Talukdar A (2006). (2006) Effective acclimatisation of *in vitro* cultured plants: methods, physiology and genetics. Floricult. Ornamental Plant Biotechnol..

[27] Ikeuchi M, Sugimoto K, Iwase A (2013). Plant Callus: mechanisms of induction and repression. Plant Cell.

[28] Folta KM (2005). Green light effects on plant growth and development. Light Sens Plants.

[29] Runkle, E. Light Wavebands and Their Effects on Plants. Preprint at https://gpnmag.com/article/light-wavebands-and-their-effects-plants/ (2015).

[30] Ngilah EI, Tsan FY, Yap BK (2018). Photoperiod and light spectrum effects on growth, pigment and ascorbic acid content of *Lactuca sativa* cv. Fire Red under controlled growth environment. Int. Food Res. J..

[31] Mawa S, Husain K, Jantan I (2013). *Ficus carica* L. (Moraceae): phytochemistry, traditional uses and biological activities. Evid.-Based Complem. Alternat. Med..

[32] Tan, H. H. Figs Are Trending In Southeast Asia Preprint at https://www.mintel.com/blog/food-market-news/figs-are-trending-in-southeast-asia. (2017).

[33] Diro M, Van Staden J (2005). The type of explant plays a determining role in the micropropagation of *Ensete ventricosum*. S. Afr. J. Bot..

[34] Gopi C, Vatsala TM, Ponmurugan P (2006). In vitro multiple shoot proliferation and plant regeneration of Vanilla planifolia Andr.-a commercial spicy orchid. J. Plant Biotechnol..

[35] Murashige T, Skoog F (1962). A revised medium for rapid growth and bioassays with tobacco tissue cultures. Physiol. Plant..

[36] Sayeed Hassan AKM, Afroz F, Jahan MAA, Khatun R (2009). *In vitro* regeneration through apical and axillary shoot proliferation of *Ficus religiosa* L.-a multi-purpose woody medicinal plant. Plant Tissue Cult. Biotechnol..

